# High-Resolution Representations Network for Single Image Dehazing

**DOI:** 10.3390/s22062257

**Published:** 2022-03-15

**Authors:** Wensheng Han, Hong Zhu, Chenghui Qi, Jingsi Li, Dengyin Zhang

**Affiliations:** 1 School of Communication and Information Engineering, Nanjing University of Posts and Telecommunications, Nanjing 210003, China; 1019010501@njupt.edu.cn (W.H.); zhu179316160@gmail.com (H.Z.); 2 School of Internet of Things, Nanjing University of Posts and Telecommunications, Nanjing 210003, China; qichenghui97@163.com (C.Q.); b19zz060605@njupt.edu.cn (J.L.)

**Keywords:** image dehazing, image restoration, deep learning, high-resolution representations

## Abstract

Deep learning-based image dehazing methods have made great progress, but there are still many problems such as inaccurate model parameter estimation and preserving spatial information in the U-Net-based architecture. To address these problems, we propose an image dehazing network based on the high-resolution network, called DeHRNet. The high-resolution network originally used for human pose estimation. In this paper, we make a simple yet effective modification to the network and apply it to image dehazing. We add a new stage to the original network to make it better for image dehazing. The newly added stage collects the feature map representations of all branches of the network by up-sampling to enhance the high-resolution representations instead of only taking the feature maps of the high-resolution branches, which makes the restored clean images more natural. The final experimental results show that DeHRNet achieves superior performance over existing dehazing methods in synthesized and natural hazy images.

## 1. Introduction

In recent years, with the development of artificial intelligence, computer vision has been applied to all aspects of our lives [1,2]. These high-level image processing tasks have high requirements for the quality of the input image. However, in hazy weather, due to many suspended particles in the air, the light scatters during the propagation process, which makes the images captured outdoors have problems such as blur and color distortion. These problems affect the subsequent image processing tasks, so the research on image dehazing is of great significance.

Image dehazing aims to recover clean images from hazy images. Early image dehazing mainly used image enhancement methods [3,4,5,6,7,8], which did not consider the cause of the image degradation but improved the contrast of the image through the method of image enhancement. The restored clean images will lose part of the image information.

Single image dehazing has made significant progress recently. Many single image dehazing methods are based on atmospheric scattering models [9,10,11], which can be expressed as

(1) I(x)=J(x)t(x) + A(1−t(x)),

where I(x) is the observed hazy image, J(x) is the real scene to be recovered, A is the global atmospheric light, and t(x) is the transmission map. If the values of t(x) and A can be estimated, the clean image J(x) can be restored.

There are two parameters to be estimated in Formula (1), so it is an indeterminate equation, and the parameters cannot be calculated by conventional methods. The early method [12,13,14] is to estimate the transmission and the global atmospheric light based on the prior knowledge of the clean images, but there are problems such as scene limitations. To solve these problems, data-driven approaches have been demonstrated to be effective. Some scholars have proposed deep learning methods [15,16,17,18,19] to estimate the transmission map, and traditional methods are used to estimate the global atmospheric light. However, these two kinds of methods have the problem of inaccurate parameter estimation.

**Figure 1:**
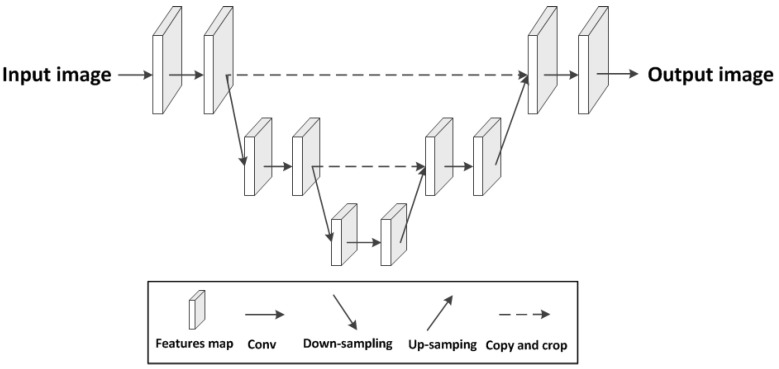
The architecture of the U-Net. The U-Net uses down-sampling to increase the receptive field and restores to the same resolution as the input image by up-sampling.

Recently, the end-to-end image dehazing method [20,21,22] provides a new idea, which inputs the hazy image into the network to obtain the clean image directly. In the end-to-end dehazing algorithm based on deep learning, a larger receptive field can extract more information in the image, to better extract the overall image and the relationship features between adjacent pixels, and better predict clean images. Most end-to-end image dehazing networks use the U-Net architecture, as shown in Figure 1, which uses down-sampling to increase the receptive field; however, this will lead to the loss of image details and overall spatial information. From the up-sampling method to recover high-resolution representations from the low-resolutions generated by high-to-low-resolution networks, it is difficult to recover such useful information, so there is the problem of image spatial information retention, which affects subsequent further processing of images. Especially in recent years, with the landing application of computer vision, high-level image processing work, such as object detection and image segmentation, has been applied to our daily production and life. Most of these high-level image processing tasks are to obtain some useful information from the input images to make corresponding judgments and decisions. If the necessary information is lost in the pre-processing of the image, it will inevitably affect the subsequent high-level processing of the image. 

To solve the problems mentioned above, in this paper, we propose DeHRNet, which is based on the high-resolution network. The high-resolution network (HRNet) [23], originally used for human pose estimation, maintains high-resolution representations by connecting branches of different resolutions in parallel and produces strong high-resolution representation by repeatedly conducting fusions across parallel convolutions. In this paper, we make a simple yet effective modification to the network and apply it to image dehazing. 

The main contributions of this paper are as follows:

1. We propose DeHRNet, which increases the receptive field of the network through parallel connections between branches of different resolutions and merges the features of branches of different resolutions at the end of each stage, effectively avoiding the issue of preserving spatial information. Moreover, we add a new stage to the original network to make it better for image dehazing. The newly added stage collects the feature map representations of all the branches of the network by up-sampling to enhance the high-resolution representations instead of only taking the feature maps of the high-resolution branches, which makes the restored clean images more natural.
2. DeHRNet is an end-to-end image dehazing network; instead of using a convolutional neural network to estimate the transmission, it effectively avoids the issue of inaccurate parameter estimation.
3. Compared with the existing methods, the network proposed in this paper has achieved good dehazing effects in both synthesized and natural hazy images.

The rest of this article is organized in the following order. In Section 2, we introduce related image dehazing algorithms. In Section 3, the method proposed in this paper is introduced. The dehazing effect and analysis of the algorithm proposed in this paper will be introduced in Section 4. Finally, in Section 5 of this paper, a summary and an outlook on image dehazing are given.

## 2. Related Work

Single image dehazing is an ill-posed problem. Prior knowledge-based and deep learning-based methods are used for single image dehazing.

The prior knowledge-based methods estimate the transmission and the global atmospheric light according to some prior knowledge of the clean images. After the statistics of a large number of clean images, He et al. [12] proposed the use of Dark Channel Prior (DCP) to estimate the transmission. Zhu et al. [13] discovered the Color Attenuation Prior (CAP). Jv et al. [14] proposed the gamma correction prior (GCP). However, the above methods have the problems of inaccurate parameter estimation, which leads to bad dehazing results. For example, since the dark channel prior is not applicable in the sky area, the image after DCP dehazing has obvious distortion in the sky area.

In recent years, many scholars have applied deep learning [24] to image dehazing and have made certain progress. Tang et al. [15] use various pre-extracted haze density-related features, such as dark channel, contrast, color difference, and saturation, and used the random forest to construct the relationship between the features and the transmission map to estimate the transmission map. Cai et al. propose DehazeNet in [16], using a convolutional neural network (CNN) to estimate the transmission map, which is divided into four stages: feature extraction, multi-scale mapping, local extremum, and nonlinear regression. Ren et al. [17] proposed a Multi-Scale CNN (MSCNN), using two convolutional neural networks, to estimate the parameters; a rough-scale network was used to estimate a rough transmission map, and then a fine-scale network was used for refinement. In [25], a Densely Connected Pyramid Dehazing Network (DCPDN) was proposed. The convolutional neural network was used to estimate the transmission map and the global atmospheric light value, respectively, and a generative adversarial network (GAN) was proposed to judge the true and false of the restored clean image and the estimated transmission map. Since the estimation of the two parameters, respectively, may increase the error, Li et al. [18] proposed the All-in-One Network (AOD-Net), combining two parameters in the atmospheric scattering model into one parameter and using CNN to estimate it. Due to the problems of the training set and the network architecture itself, the dehazing algorithm based on deep learning has a certain deviation in the estimation of the transmission map, which makes it impossible to obtain a satisfactory dehazing effect for some scenes when dehazing the real hazy image. Unlike some of the abovementioned methods that use CNN to estimate the transmission, Liao et al. [20] used CNN to predict the haze density of the image and then subtracted the predicted haze density from the hazy image to obtain a clean image. Dong et al. [21] proposed a Multi-Scale Boosted Dehazing Network with Dense Feature Fusion (MSBDN). The network is designed based on the two principles of boosting and error feedback. The clean image is restored by adding the Strength–Operate–Subtract strategy to the decoder of the model, and the back-projection feedback solution is used to solve the problem of spatial information retention. In [22], a Cycle-Dehaze end-to-end network is proposed, the network does not require pairs of hazy/clean images to train the network Shao et al. [26] proposed a domain adaptation framework called DA-dahazing, which consists of an image transformation module and two image dehazing modules, which first transforms the input hazy image from one domain to another, and then takes the transformed image and the original image as input to dehaze the image. Wu et al. [27] proposed novel contrast regularization (CR) technology based on contrast learning that uses the information of the hazy image and the clear image as negative and positive samples, respectively. 

Based on the above analysis, in this paper, we proposed an end-to-end image dehazing network called DeHRNet. DeHRNet maintains high-resolution representations through the whole process by connecting different resolution branches parallel, and the feature maps of different branches will be fused to produce a rich high-resolution representation.

## 3. The Proposed DeHRNet

**Figure 2:**
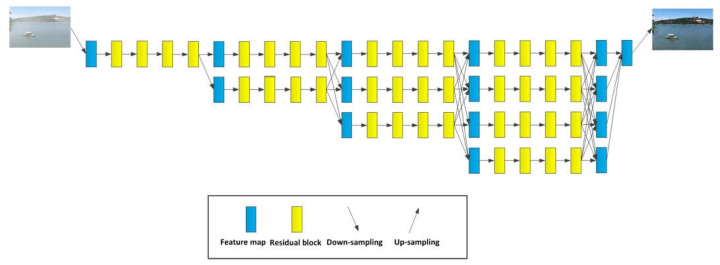
The architecture of the DeHRNet. Parallel connection between different branches to ensure the same resolution.

Based on the high-resolution network (HRNet), this paper proposes an end-to-end image dehazing network, called DeHRNet. Different from the network used in the existing end-to-end dehazing methods, DeHRNet starts from a high-resolution branch as the first stage and gradually adds a low-resolution branch at the beginning of each stage and connects all branches in parallel. At the end of each stage, we conduct fusions across the parallel branches. The difference from the original HRNet is that we add a new stage to the original network to make it better for image dehazing. The newly added stage collects the feature map representations of all branches of the network by up-sampling to enhance the high-resolution representations instead of only taking the feature maps of the high-resolution branches, which makes the restored clean images more natural. Although a small amount of calculation has been added, good results have been obtained. Figure 2 is the architecture of the network. We detail the architecture and the improvement of the original network as follows.

### 3.1. Detailed Network Structure

The input hazy image first undergoes a 3 × 3 down-sampling with a step size of 2, which is mainly used for feature extraction of the input hazy image and reducing the calculation amount of the subsequent process, and then enter the first stage of the network. Starting from the first stage of the network, at each stage the network generates a new low-resolution branch based on the previous stage.

The structure of the first, second, third, and fourth stages of the network is roughly the same, and the second stage is used to explain. To explain the relationship between the stages and branches of the network, the following formula is given:

(2) $$\begin{array}{c} F\to F_{11}\to F_{21}\;\to F_{31}\;\to F_{41}\\ \;\;\;\;\;\;\;\;\;\;\;\;\;\;\;↘F_{22}\;\to F_{32}\;\to F_{42}\\ \;\;\;\;\;\;\;\;\;\;\;\;\;\;\;\;\;\;\;\;\;\;\;\;\;\;\;↘F_{33}\;\;\to F_{43}\\ \;\;\;\;\;\;\;\;\;\;\;\;\;\;\;\;\;\;\;\;\;\;\;\;\;\;\;\;\;\;\;\;\;\;\;\;\;\;\;\;\;↘F_{44}, \end{array}$$

where, among them, F is the input of feature maps. $F_{ij}$ is the i-th stage and j-th branch feature maps.

**Figure 3:**
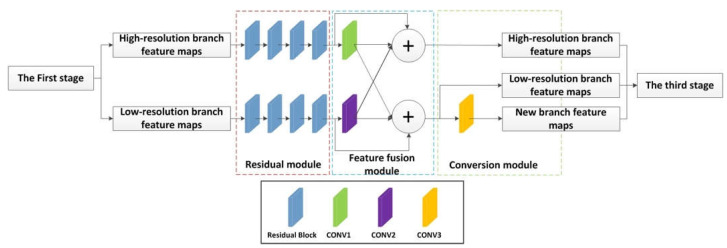
The overall structure of the second stage. Each stage is composed of a residual module, a feature fusion module, and a conversion module.

Figure 3 shows the overall structure of the second stage, and Table 1 shows the parameters in the network of the second stage. In Table 1, the “Conv1”, “Conv2”, and “Conv3” correspond to Figure 3’s “Conv1”, “Conv2”, and “Conv3”, respectively. Conv1 is used to down-sample the feature map of the branch with a higher resolution, so that the resolution of the feature map is consistent with the resolution of the low-resolution branch, which is convenient for feature fusion. Similarly, conv2 is up-sampling. Conv3 is down-sampling to generate a new resolution branch. Each stage is composed of a residual module, a feature fusion module, and a conversion module. The output of the residual module is connected to the feature fusion module, and the output of the feature fusion module is used as the input of the conversion module. 

**Figure 4:**
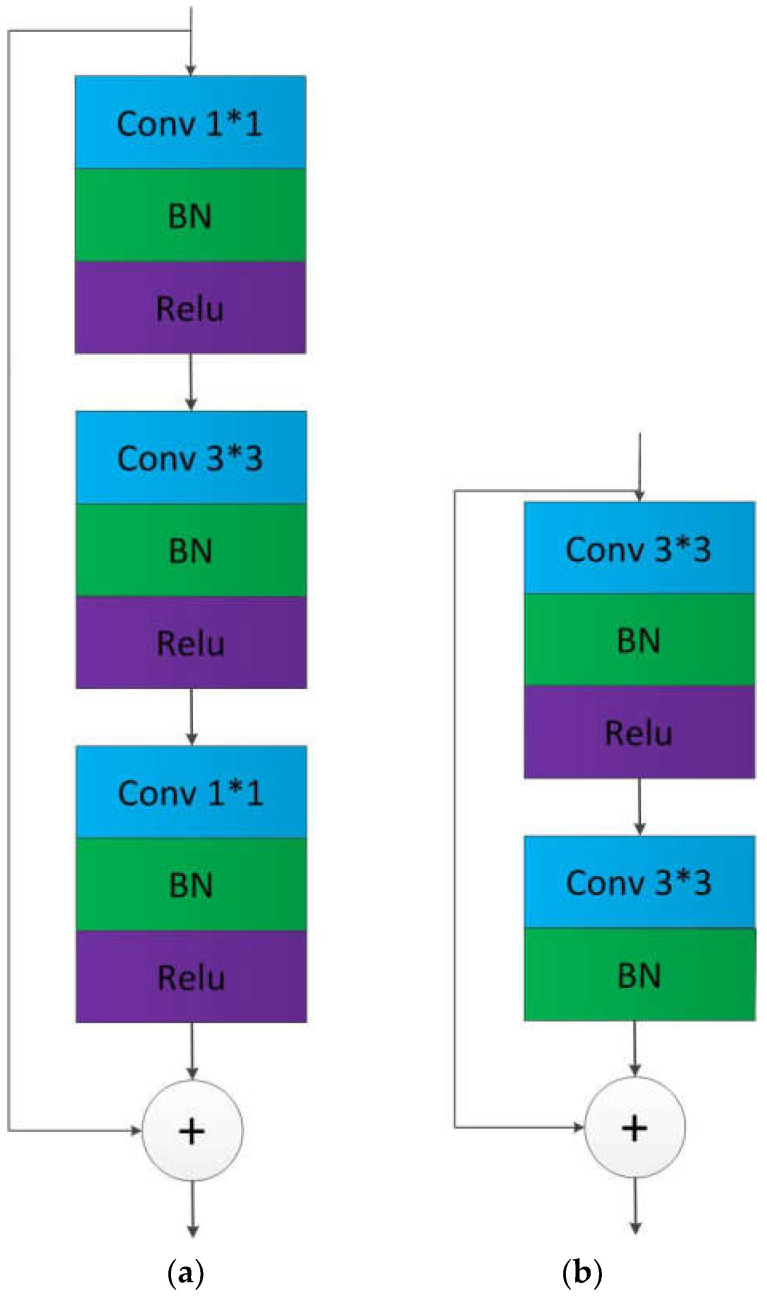
The architecture of the residual block. (**a**) The residual structure of the first stage. (**b**) The residual structure of the second, third, and fourth stages. Conv means a convolution operation, and k×k behind it means convolution kernel size. BN means batch normalization. Relu means activation function.

The residual module consists of four residual networks. The residual structure of the first stage of the network is shown in the left figure of Figure 4, which consists of two 1×1 convolutions, a 3×3 convolution, and a skip layer connection, and the convolution step size is 1. The residual structure of the second, third, and fourth stages of the network are shown in the right figure of Figure 4, which consists of two 3×3 convolution kernels and one skip layer, and the convolution stride is 1.

The feature fusion module includes up-sampling and down-sampling. In the up-sampling process, the feature map of the low-resolution branch is passed through a 1×1 convolution to make the number of channels of the low-resolution feature map consistent with the number of channels of the high-resolution feature map, then converted to the same resolution by the nearest neighbor interpolation method, and finally adding the feature maps of the two branches. In the down-sampling process, the feature map of the high-resolution branch is firstly convolved with a stride of 2 by 3×3 to convert it into a feature map with the same resolution and the same number of channels as the low-resolution branch, and finally, the two are added up. Through the above mentioned up-sampling and down-sampling, the feature fusion between different resolution branches is completed, which is conducive to the exchange of learned information between the different branches.

The last module is the conversion module. Through the conversion module, the network adds a new resolution branch based on the previous stage, and the resolution of the new branch is half of the resolution of the feature map of the low-resolution branch of the previous stage, and the number of channels is twice. In the conversion module of Figure 3, a 3×3 convolution with a stride of 2 is used to make the new branch resolution half of the original branch resolution. At the same time, by adjusting the number of convolution kernels, the number of channels is twice the original number of channels.

**Figure 5:**
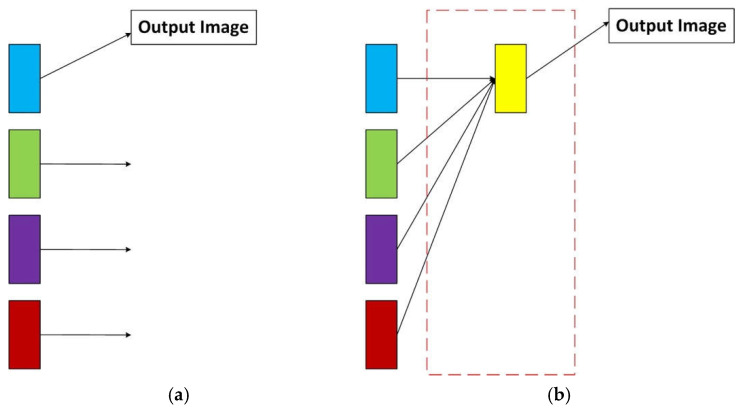
Network comparison. (**a**) The original network. (**b**) The newly added stage. The upward arrow is up-sampling, and the rectangles of different colors represent feature maps with different resolutions. In (**b**), the part framed by the red dotted line is the newly added stage.

We add a new stage to the original HRNet to make it better for image dehazing work. In the original network, only feature maps from the high-resolution branch are considered as outputs, as shown in the left panel of Figure 5. This means that feature maps from low-resolution branches are lost. We make a simple and effective modification to fuse the feature maps from the low-resolution branch with the feature maps from the high-resolution branch instead of only using feature maps from the high-resolution branch, to make full use of the feature information extracted from different resolution branches, resulting in a rich high-resolution branch, as shown on the right in Figure 5. The improved boost will be discussed in later sections. We first keep the resolution of low-resolution feature maps consistent with the number of channels of high-resolution feature maps by up-sampling, and then employ bilinear interpolation to rescale the feature maps of the low-resolution branch to high-resolution. Since bilinear interpolation considers the pixel values of four points in the image, its calculation is more complicated than nearest neighbor interpolation, but its effect is better. Considering that the clean image will be output after this stage, there are high requirements for the result, so bilinear interpolation is selected here and in the previous stage of the network, because the convolutional neural network is mainly used to extract the features; so, the nearest neighbor interpolation, which is relatively simple to calculate, is selected. Then, we rescale the feature map to the same size as the input hazy image again through bilinear interpolation, to directly obtain the clean image after dehazing. Although the calculation amount is slightly increased, the final dehazing effect is more natural.

### 3.2. Loss Function

The loss function$\;L_{2}$ used in training the network in this paper can be expressed as

(3) $$L_{2}=\frac{1}{N\times H\times W}\overset{N}{\underset{i=1}{\sum }}{\left|\left|J_{i}-J_{i}^{*}\right|\right|}^{2},$$

where, among them, $J_{i}^{*}$ is the dehazed image restored by the network, $J_{i}$ is the real clean image, H and W are the height and width of the image, and N is the number of images in a batch.

## 4. Results

In this section, we will first introduce the relevant parameters and details of the experiment, and then compare it with the existing methods on the synthetic data set and the real-world data set. For the comparison of the synthetic data sets, we use a combination of subjective and objective methods for comparison. Objective comparison methods all use the peak signal-to-noise ratio (PSNR) and structural similarity index (SSIM) [28], two indicators for comparison. Since the real-world data sets do not have real clean images, only subjective evaluations are made.

### 4.1. Database

In the training process of deep learning, the quality of the network training depends to a large extent on the quality of the training set, but it is difficult to obtain a real hazy/clean image pair. Earlier people used the NYU dataset as a training set. Since the NYU dataset contains real clean images and their corresponding transmission map, the hazy images corresponding to the clean images in the dataset are artificially synthesized through the atmospheric scattering model formula. However, since the NYU dataset only contains indoor images, not outdoor images, and the hazy images synthesized according to the atmospheric scattering model formula are largely different from the real hazy images, the training effect of the later network is not good. Since it is difficult to obtain hazy/clean image pairs in the real world, the RESIDE dataset [29] is used to train the network. The dataset contains both indoor and outdoor hazy/clean image pairs. The outdoor training set (OTS) contains 2061 clean images and 72,135 hazy images; that is, an outside clean image generates 35 hazy images with different haze densities. As a result, we randomly select 2061 clean images from OTS as the training set. An image is randomly selected from the hazy image corresponding to the clean image to form a hazy/clean image pair for network training. The synthetic objective test set (SOTS) contains 500 outdoor synthetic hazy images, and we used SOTS as a test set to test our network.

### 4.2. Experiment Environment

The deep learning framework used in the network in this paper is Pytorch. The size of the input image to the network is 550 × 400. The batch size was set to 12, the number of training iterations was 484, the initial learning rate of the network was 0.0001, the stochastic gradient descent algorithm (SGD) was used to optimize the network parameters, and a NVIDIA GeForce RTX 3090 was used to train the network.

### 4.3. Result and Analysis

In this section, the dehazing algorithm proposed in this paper is compared objectively and subjectively with other mainstream dehazing algorithms. Other mainstream methods include: Dark Channel Prior (DCP) [12], Color Attenuation Prior (CAP) [13], and DehazeNet [16], AOD-Net [18], MSBDN [21] and DA-hazing [26]. At the same time, we select several real hazy images that are commonly used in the evaluation of image dehazing algorithms as the subjective evaluation basis.

#### 4.3.1. Experimental Results and Analysis on the Synthetic Hazy Image Test Set

**Figure 6:**
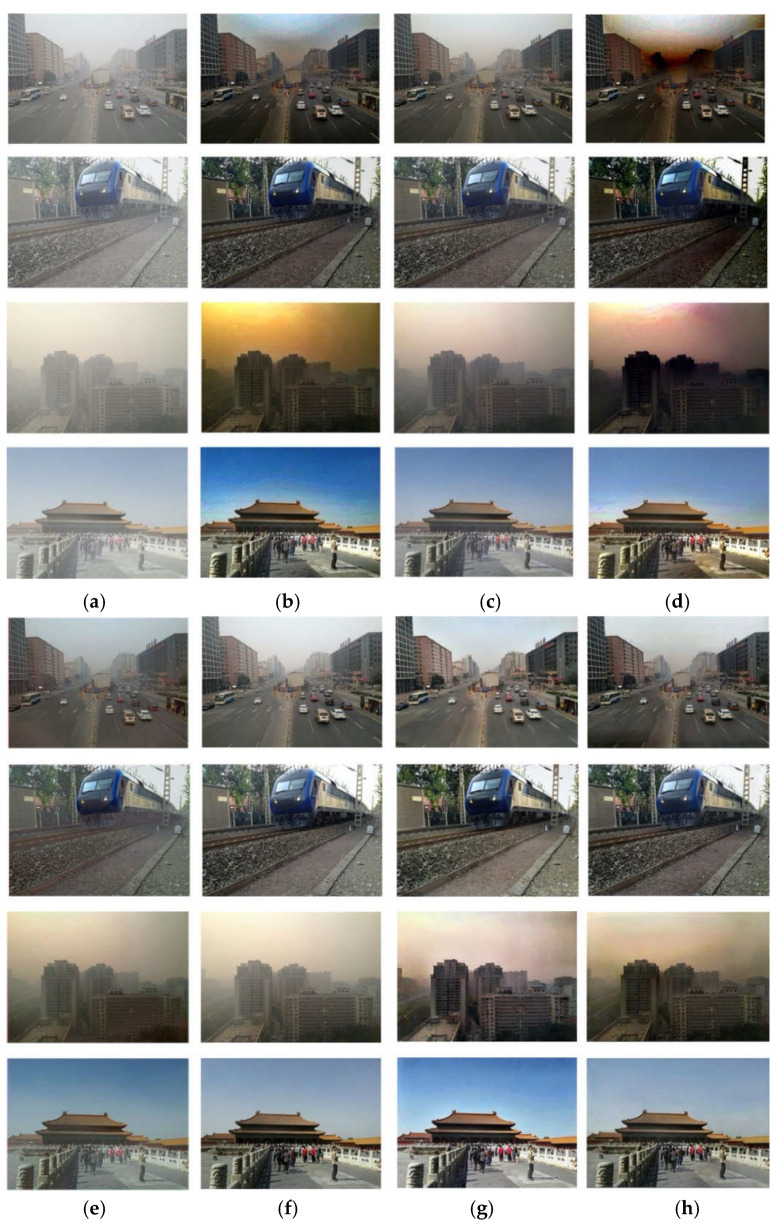
Dehazing effect of different dehazing algorithms on the SOTS dataset. (**a**) Input image; (**b**) DCP [12]; (**c**) CAP [13]; (**d**) DehazeNet [16]; (**e**) AOD-Net [18]; (**f**) MSBDN [21]; (**g**) DA-dahazing [26]; (**h**) Ours.

To verify the effectiveness of the dehazing model DeHRNet proposed in this paper, we conducted experiments with hazy images in the RESIDE dataset and compare and analyzed the effect of the algorithm proposed in this paper with the mainstream dehazing algorithm on image dehazing. The input images of all algorithms were from the RESIDE dataset SOTS Outdoor synthetic hazy images, for different scenes. Table 2 shows the differences in the objective evaluation indicators of the different dehazing algorithms. Figure 6 shows the dehazing effect of various mainstream dehazing algorithms on synthetic datasets.

To better explain Table 2, we first introduce the two indicators in Table 2. PSNR is often used as the main indicator for image quality evaluation, and its definition is based on the mean square error (MSE). Given a hazy image I and a clean image J of size *H* × *W*, the mean square error is defined as

(4) $$\mathrm{MSE}\;=\;\frac{1}{H\times W}\sum_{i=0}^{H-1}\sum_{j=0}^{W-1}{\;[I\left(i,j\right)-J\left(i,j\right)]}^{2},$$

Among them, I(i,j) is the pixel value of the hazy image at the pixel point (i,j), and J(i,j) is the pixel value of the clean image at the pixel point (i,j). The definition of PSNR obtained from Formula (4) is as follows:

(5) $$\mathrm{PSNR}\;=\;10log_{10}\left(\frac{{\left(maxI\right)}^{2}}{MSE}\right)\;=\;20\times log_{10}\left(\frac{maxI}{\sqrt{MSE}}\right),$$

Among them, maxI is the maximum value of the image pixel. In general, the larger the value of PSNR, the better the image quality.

SSIM [28] is a measure of how similar two images are. When used as a dehazing evaluation index, generally one is the image after the algorithm is dehazed, and the other is the corresponding original hazy image. In general, the larger the value of SSIM, the more the structural information and the better the quality of the restored clean image.

Table 2 shows the differences in the objective evaluation indicators of the different dehazing algorithms. It can be seen from Table 2 that the algorithms based on physical models [12,13] are less effective, the non-end-to-end algorithms based on deep learning perform better than algorithms based on physical models, and the end-to-end algorithms based on deep learning work best. The dehazing algorithm proposed in this paper is an end-to-end algorithm based on deep learning and better than most dehazing algorithms in two objective evaluation indicators, which also objectively proves the effectiveness of the dehazing algorithm proposed in this paper. In addition, compared with the original network, the improved dehazing algorithm in this paper has obvious improvements in two indicators. To further prove the effectiveness of the algorithm proposed in this paper, the next part of this section will give the dehazing effect of the algorithm proposed in this paper on real hazy images.

Figure 6 shows the dehazing effect of different dehazing algorithms on the outdoor synthetic dataset of the SOTS subset of the RESIDE dataset. As can be seen from Figure 6, the main problem of DCP [12] is that the recovered clean image will have obvious color distortion, especially in its sky part, mainly because the dark channel prior knowledge is not applicable in the sky region. The main problem of CAP [13] is the incomplete dehazing, as shown in the third row of Figure 6c. DehazeNet [16] handles the light haze in the image well, but when the haze in the image is thick, the restored clean image has obvious color distortion problems, and the image is dark as a whole. AOD-Net [18] also has the problem of incomplete dehazing, as shown in the second and fourth lines of Figure 6e. Compared with the previous several dehazing algorithms, MSBDN [21] performs better, but its dehazing effect is not good when there is haze in the distant image, as shown in the first row of Figure 6f. The clean image restored by DA-dahazing [26] is generally brighter than the real clean image, as shown in the first and fourth rows of Figure 6g, and there is color distortion in the sky in the fourth row of Figure 6g. In general, the dehazing algorithm proposed in this paper can achieve a satisfactory dehazing effect in both objective evaluation indicators PSNR and SSIM, as well as in the mist and dense haze, close-range, and long-range scenes under subjective evaluation.

#### 4.3.2. Experimental Results and Analysis on the Real Hazy Image Test Set

In order to verify the effectiveness of the dehazing algorithm proposed in this paper on real hazy images, this section selects several typical real-world hazy images for experiments, and compares them with the mainstream dehazing algorithms DCP [12], CAP [13], DehazeNet [16], AOD-Net [18], MSBDN [21] and DA-dahazing [26]. 

**Figure 7:**
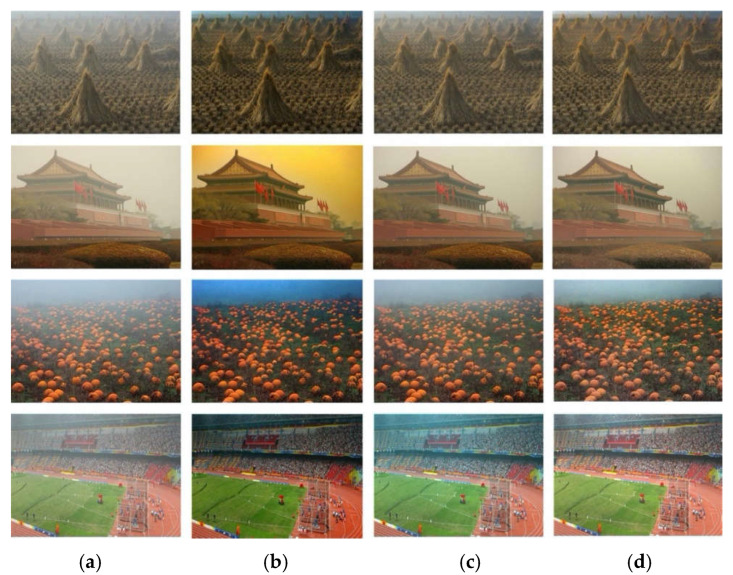

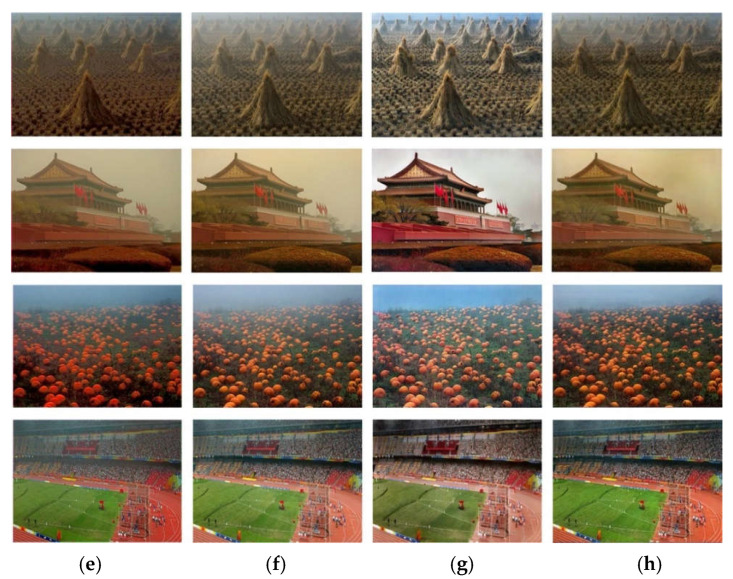
Dehazing effect of different dehazing algorithms on real-world images. (**a**) Input image; (**b**) DCP [12]; (**c**) CAP [13]; (**d**) DehazeNet [16]; (**e**) AOD-Net [18]; (**f**) MSBDN [21]; (**g**) DA-dahazing [26]; (**h**) Ours.

The real hazy image comes from the real world, not the artificially synthesized hazy image. The haze concentration is unevenly distributed in the whole image and has great uncertainty. In Figure 7, due to the applicability of prior knowledge, the dehazing effect of DCP [12] in the sky part is irrational. The sky part in the second row in Figure 7b has obvious color distortion problems, and the restored clean image has high contrast and is dark overall, as shown in the first, fourth, and fourth rows in Figure 7b. In the CAP [13] algorithm, the image after dehazing is bright and there is a problem of incomplete dehazing, as shown in the third and fourth rows in Figure 7c. The above two dehazing algorithms are based on prior knowledge, but the prior knowledge is not applicable in some scenarios, and the dehazing algorithm proposed in this paper is an end-to-end dehazing algorithm based on deep learning, so there are no such problems. For the above problems, a good dehazing effect can be achieved for hazy images in most scenes. As shown in Figure 7d, the part of the flower bed in front of the second row has obvious color distortion, and the image dehazed by DehazeNet [16] has obvious color distortion and loss of details. In the AOD-Net [18] algorithm, the dehazed image has high contrast and is dark overall, as shown in the third and fourth rows in Figure 7f. The above two dehazing algorithms are algorithms for estimating parameters based on deep learning. However, due to the rationality of the network architecture and formulas, there are inaccurate parameter estimation problems in some scenarios. The dehazing algorithm proposed in this paper is an end-to-end dehazing algorithm based on deep learning and does not estimate any formula parameters, so the above problems do not exist. The end-to-end dehazing algorithm DA-dahazing [26] based on deep learning, the image after dehazing is bright as a whole and has some color distortion problems, as shown in Figure 7g in the distance in the first row and the second row. For the sky part, as well as the stadium part in the fourth row, the restored images do not meet the requirements of human vision. Relatively speaking, the clean images recovered by the dehazing algorithm proposed in this paper are clearer and more natural and meet the requirements of human vision. In conclusion, the algorithm proposed in this paper can also achieve a satisfactory dehazing effect in real hazy images.

#### 4.3.3. Dehazing Time Analysis of a Single Image

In this section, we will compare and analyze the dehazing time of several deep learning-based dehazing algorithms for a single image. The specific results are shown in Table 3. All input images are from the RESIDE dataset SOTS subset outdoor test set, except for DA-dahazing [26] (input image size is 640 × 480), MSBDN [21] (due to network reasons, the width and height of the input image need to be divisible by 16, the image size of the input is 608 × 448), and the size of other network input images is 550 × 400. Except for the implementation framework of DehazeNet being Caffe, the implementation frameworks based on the deep learning algorithms are Pytorch. This subsection first calculates the dehazing time for all images in the dataset, including the model loading time, and then divides it by the total number of images to get the dehazing time for a single image. It can be seen from Table 3 that although the parameters of the improved network increase, the single image dehazing time of the dehazing algorithm proposed in this paper is better than DehazeNet [16], AOD-Net [18], and MSBDN [21], second only to DA-dahazing [26].

## 5. Conclusions

In this paper, we make a simple modification to the high-resolution representation network and apply the improved network to image dehazing. Different from the network used in the existing end-to-end dehazing methods, DeHRNet starts from a high-resolution branch as the first stage gradually adds a low-resolution branch at the beginning of each stage and connects all branches in parallel. At the end of each stage, we conduct fusions across parallel branches. The difference from the original HRNet is that we add a new stage to collect the feature map representations of all branches of the network by up-sampling to enhance the high-resolution representations instead of only taking the feature maps of the high-resolution branches. The final experimental results show that the restored clean image obtained by DeHRNet is clearer and natural.

Although we successfully applied the improved network for image dehazing, there is still some work to be done; that is, although the dehazing time for a single image is short, the network is relatively complex and has a few more parameters. Nowadays, lightweight networks become mainstream; so, in the future, we will further research network lightweighting. For example, we can use depthwise separable convolution to replace the ordinary standard convolution in the network to reduce the network parameters and further improve the performance of the network. Moreover, since it is difficult to obtain hazy/clean image pairs in the real world, unsupervised learning methods may achieve better dehazing results. We will carry out this research in the future.

## Figures and Tables

**Table 1:** The parameters in the network of the second stage. Conv means a convolution operation, and “Conv1”, “Conv2”, and “Conv3” correspond to Figure 3’s “Conv1”, “Conv2”, and “Conv3”, respectively.

Type	Input Image Channel Number	Filter	Filter Number	Stride
Conv1	32	3×3	64	2
Conv2	32	3×3	32	1
Conv3	64	3×3	128	2

**Table 2:** Comparison of our algorithm and other algorithms on the objective indicators of the dehazing results on the SOTS dataset. Bold texts are the best and our experimental results.

Algorithm	Evaluation Indicators	
	PSNR	SSIM [28]
DCP [12]	16.94	0.8548
CAP [13]	22.30	0.9053
DehazeNet [16]	22.31	0.6828
AOD-Net [18]	19.58	0.8390
MSBDN [21]	**30.61**	**0.9396**
DA-dahazing [26]	22.84	0.8845
The original network	20.97	0.7523
Ours	**23.15**	**0.8013**

**Table 3:** Comparison of single image dehazing time for the different dehazing algorithms. Bold texts are the best and our experimental results.

Algorithms	DehazeNet	AOD-Net	MSBDN	DA-Dahazing	Our
**Time (s)**	4.93	3.24	0.47	**0.17**	**0.40**

## Data Availability

Data sharing is not applicable to this article.
